# The Role of Plant-Based Diets for Cancer Survivors and Planetary Health

**DOI:** 10.3390/curroncol33020072

**Published:** 2026-01-26

**Authors:** Kaitlyn H. Kwok, Thomas E. Hedley, Caroline J. Mariano

**Affiliations:** 1 Department of Family Medicine, University of British Columbia, Vancouver, BC V5Z 4E6, Canada; 2 Vancouver Department of Medical Oncology, BC Cancer Agency, Vancouver, BC V5Z 4E6, Canada

**Keywords:** planetary health, survivorship, nutrition, plant-based diets

## Abstract

Purpose: A growing body of evidence has emerged on the role of diet for health outcomes in cancer survivors. Patients transitioning to post-treatment care may seek guidance on dietary changes, and summaries of the evidence for dietary patterns recommended by guidelines can support providers in effectively answering questions. Increasing evidence suggests that food choices impact planetary health. Plant-based diets are one eating pattern that may improve patient outcomes and planetary health. Methods: We performed a literature review and used narrative reporting to summarize evidence for plant-based diets and offer specific guidance for breast, colorectal, and prostate cancer patients who are post-diagnosis. Specifically, we reviewed impacts on recurrence, all-cause, and cancer-specific mortality. Results: Increased fibre intake by consuming foods like fruits, vegetables, and whole grains is associated with a decreased risk of breast cancer-specific and all-cause mortality, as well as reduced colon cancer-specific mortality. Replacing refined grains with whole grains is associated with improved disease-free survival for colon cancer survivors. Higher tree nut consumption is associated with improved disease-free survival for breast, colorectal, and prostate cancer survivors. Soy is safe to consume for breast cancer survivors and is associated with a reduced risk of recurrence. Conversely, more Western dietary patterns high in processed meat intake are associated with an increased risk of colon cancer recurrence and prostate cancer mortality. There are also environmental benefits of a shift towards plant-based diets to address the adverse health outcomes associated with climate change and its potential impact on cancer care delivery as previously outlined in a 2024 ASCO policy statement. Conclusions: Based on the best existing evidence, providers can suggest that patients consider plant-based dietary patterns in the post-treatment phase of their cancer care to support health outcomes and planetary health.

## Simple Summary

Many cancer survivors ask for guidance on what foods to eat to support their health after treatment. The role of diet in cancer survivorship is a growing area of research. This article summarizes current evidence on dietary patterns associated with health outcomes in cancer survivors, with a focus on plant-based diets. Evidence suggests that dietary patterns high in fruits, vegetables, whole grains, legumes, nuts, and soy foods, and low in red and processed meats and refined grains, may be associated with improved outcomes after a diagnosis of breast, colorectal, or prostate cancer. The article also offers guidance on food groups that may be prioritized or limited based on cancer type. In addition, the potential environmental benefits of plant-based dietary patterns are discussed, highlighting links between food choices and personal and planetary health.

## 1. Introduction

In Canada, the number of cancer patients in the survivorship portion of their cancer journey continues to grow as mortality rates decline [1]. Based on data from 2015 to 2017, the predicted 5-year net survival rate of all cancers combined was 64%, with the highest survival rates among individuals diagnosed with breast cancer (89%) and prostate cancer (91%) [2]. As more patients continue to enter post-treatment care, a growing interest has developed in how lifestyle measures can improve long-term health for cancer survivors.

Healthcare providers play a critical role in guiding patients through survivorship, addressing concerns related to treatment, side effects, and alternative therapies. Following initial diagnosis, patients often seek guidance on lifestyle modifications, including dietary adjustments and changes in physical activity. Primary concerns centre on ways to prevent disease progression and minimize the likelihood of recurrence. Balancing discussions regarding lifestyle recommendations with other aspects of patient care can be challenging, particularly given a rapidly evolving body of evidence and limitations in time. Thus, we thought it pertinent to review some of the existing evidence for post-diagnosis dietary patterns and outcomes to provide guidance to providers.

Both the Canadian Cancer Society (CCS) and the American Cancer Society (ACS) suggest a healthful eating pattern after a cancer diagnosis. The CCS and ACS similarly describe a healthful diet as being rich in foods derived from plants, including fruits, vegetables, whole grains, legumes, nuts, and seeds. The CCS outlines a plant-based diet where at least three-quarters of each meal is composed of plant foods, including fruits, vegetables, and whole grains [3,4]. An example of this plant-based dietary pattern is the Mediterranean diet [4], which encourages limited intake of animal-derived products such as meat, fish, dairy, and eggs but does not mandate strict adherence to vegetarianism or veganism. In summary, a plant-based dietary pattern offers a flexible approach that allows for the occasional inclusion of animal products if desired, while maintaining focus on plant-based foods as the primary component of meals.

In this paper, we review some of the evidence for dietary interventions for patients who have previously been diagnosed with cancer, specifically colorectal (CRC), breast, and prostate cancer, for which survivorship rates are the highest and there is the most available evidence [2]. We focus on evidence commenting on cancer mortality, overall mortality, and risk of recurrence. We also briefly mention some of the proposed pathophysiologic mechanisms. Furthermore, as another emerging issue in oncology care is the impact of climate change on both human and planetary health, we acknowledge the environmental co-benefits of a plant-based diet.

## 2. Methods

We performed a literature review and, using narrative reporting, synthesized the evidence on plant-based diets in cancer survivors, as recommended in the existing guidelines from the CCS and ACS. A formal systematic review was not performed due to the heterogeneity of studies, including different tumour types and interventions we sought to capture. Searches were performed through the BC Cancer Clinical Library and included the search engines EBSCO (CINAHL), OVID (Medline, EMBASE, EBM Reviews), and Google Scholar. Publications over a ten-year period, from 2015 to 2025, were included. Only papers published in English were included in the searches. Papers were subsequently excluded if they did not apply to patients in the post-diagnosis setting. We also excluded papers where outcomes were not recurrence, disease-specific, or overall survival.

Combinations of search terms for plant-based diets such as “vegetarian diet,” “vegan diet,” “lactovegetarian diet,” “ovovegetarian diet”, and other variations were included in the search criteria to generate more inclusive results and include studies whereby a dietary intervention would limit or restrict animal-based proteins in favour of other alternatives. Most studies have classified diets into categories, such as a “healthful diet” versus an “unhealthful diet” or used scoring indices such as the Healthy Eating Index 2005 (HEI-2005) [5,6,7]. The typical profile for these healthful diets included a plant-based approach high in fruits, vegetables, and whole grains supplemented by fish, dairy products, and white meat, while avoiding red meat, processed meat, and high-caloric beverages [7]. Other studies included a plant-based dietary index (PDI) to examine adherence to a plant-based eating pattern, which scored and quantified healthy plant foods positively, such as fruits and vegetables, whereas less healthy plant-based food and animal products scored negatively [5,8,9]. For the purposes of this paper, we adopted the language of “plant-based diet” (PBD) in comparison to a “non-plant-based diet” (nPBD) to describe the opposing diet spectrums relative to each other.

In our synthesis, we found 30 studies that discussed multiple cancers, 15 breast cancer-specific, 10 colon cancer-specific, and 6 prostate cancer-specific. A total of 15 papers included some discussion of environmental co-benefits. For the purposes of this review, we summarized seminal papers documenting environmental and climate benefits of plant-based versus animal protein-based diets.

## 3. Evidence for Plant-Based Diets

Plant-based diets focus on the consumption of foods derived from plants, such as fruits, vegetables, whole grains, legumes, nuts, and seeds, while limiting red and processed meats. The ACS guidelines highlight a 2020 review and meta-analysis by Molina-Montes et al., which evaluated the impact of plant-based dietary patterns on cancer outcomes. When considering vegan, vegetarian, and Mediterranean dietary patterns in the pre-diagnosis setting, only closer adherence to a Mediterranean dietary pattern was associated with lower cancer-related mortality. In the post-diagnosis setting, they advised that there was not a sufficient number of studies with clearly defined plant-based diets to conclude their impact on cancer outcomes [10]. Following these guidelines, a 2023 meta-analysis by Trauchburg et al. demonstrated that cancer survivors consuming the most versus the least healthful diets post-diagnosis had significantly lower risks of all-cause and cancer-specific mortality. The typical profile for these healthful diets was similar to the plant-based approach outlined by the CCS and ACS, high in fruits, vegetables, and whole grains supplemented by fish, dairy products, and white meat, while avoiding red meat, processed meat, and high-caloric beverages [7]. To organize the evidence in this review, we opted to categorize each food group separately below and pair them to suggested pathophysiologic mechanisms, and compare quantities used in individual studies with those recommended in the existing guidelines where available.

### 3.1. Fruits and Vegetables

Fruits and vegetables play a key role in the basis of a plant-based diet and act as a rich source of antioxidants and fibre [4,7]. The American Cancer Society recommends increasing the intake of whole fruits in a variety of colours and leafy greens for individuals diagnosed with cancer [4]. Diets rich in fruits and vegetables have previously been studied in relation to cardiovascular health, such as within the Mediterranean and the Dietary Approaches to Stop Hypertension (DASH) diets. The fibre found in fruits and vegetables may also have protective properties against colorectal cancer [11,12,13]. There are several mechanisms thought to be implicated in the anti-carcinogenic properties of fibre found in fruits and vegetables. These include quicker transit time through the bowel, reduced production of carcinogenic metabolites, and tumour-suppressive effects via fermentation [13,14]. Fibre facilitates fermentation in the gut, which produces integral short-chain fatty acids such as butyrate, acetate, and propionate, which can prevent cell proliferation and increase apoptosis [13,14].

One systematic review by Hardt et al. in 2022 discussed that in a prospective cohort study of 992 stage III CRC patients, consuming five or more servings of fruits and vegetables per day had a 40% lower risk of mortality compared with those consuming less than five servings per day [6,15]. As per the supplementary material for Van Blarigan’s study, five servings were defined as 2.5 cups of vegetables and fruits [15]. An RCT performed by Nordengen included patients with stage I–III CRC who were post-surgery and promoted a PBD including the daily intake of fruits and vegetables ≥ 500 g (2 cups) [16]. At one year, they found a 32% reduction in DNA base oxidation in the dietary group compared to the control group, although the clinical significance of this is still undetermined [16].

While other studies do not specify exact quantities of fruits and vegetables, in the meta-analysis performed by Etesami et al. in 2025, higher proportions of “healthful” plant-based foods within a PBD were associated with a reduced risk of both overall mortality and prostate cancer mortality [17]. In regard to breast cancer, as per the systematic review by Hardt et al., the two included meta-analyses examining fruit and vegetable intake [18,19] showed no association between fruit and vegetable intake with the overall survival of breast cancer; however, in Nurses’ Health Studies [20], individuals with a higher cumulative average of fruit and vegetable intake had a decreased relative risk of all-cause mortality [6]. Consequently, we suggest that prostate and breast cancer patients prioritize fruits and vegetables in their diets to support fibre intake and possibly decrease mortality risk, but the quantities required for these benefits are difficult to establish.

Lastly, lycopene, an antioxidant found in plants, especially tomatoes, has been explored for its impact on prostate cancer incidence and outcomes [21]. A large cohort study showed that post-diagnosis dietary lycopene levels as well as tomato consumption were not associated with improved prostate cancer-specific mortality. However, among patients with high-risk prostate cancer, those consuming higher levels of dietary lycopene had a decreased risk of prostate cancer-specific mortality compared to those with lower intake [22]. While there is very limited evidence for this, prostate cancer survivors with high-risk disease may consider the daily consumption of lycopene-rich foods.

### 3.2. Whole Grains

Whole grain foods consist of the entire grain, including three parts: the bran, germ, and endosperm. Quinoa, oatmeal, and brown rice are all examples of whole grain foods. They are considered healthier than refined grains, which are lower in fibre, vitamins, and minerals due to the removal of the bran and germ components of grains [23]. For breast cancer, although there is no direct link between whole grains and prognosis, a dose–response meta-analysis of prospective cohort studies showed that higher fibre intake is associated with a decreased risk of breast cancer-specific and all-cause mortality [24].

The evidence for consuming whole grains within a PBD has primarily been observed in colorectal cancer survivors. In a systematic review and meta-analysis by Hoang et al. focused on colorectal cancer, sub-group analysis showed that higher whole grain intake post-diagnosis was associated with a significantly decreased risk of colorectal cancer-specific and all-cause mortality [25]. Similarly, a prospective observational study by Song et al., including 1575 participants with stage I–III colorectal cancer, found that each 20 g per day increase in whole grain intake was associated with a decreased risk of colorectal cancer-specific mortality [26]. Within this study, cereal fibre, which is abundant in whole grains, was associated with a lower risk of colorectal cancer-specific and all-cause mortality. In regard to quantity, post-diagnosis fibre intake of 24 g per day was the level found to maximally reduce the risk of all-cause mortality [26]. In a dose–response meta-analysis of prospective cohort studies, Zhao et al. observed that a maximum daily dietary fibre intake of 22 g of fibre was associated with the greatest reduction in the risk of colorectal cancer mortality [27]. Furthermore, a prospective observational study by Brown et al. of 1024 patients with stage III colon cancer demonstrated that substituting one daily serving of refined grains to whole grains was associated with a 13% lower relative risk of recurrence and mortality [28].

One possible explanation for improved colorectal cancer prognosis is that whole grain foods in general serve as a source of antioxidants and phytoestrogens, similar to fruits and vegetables. As described above, when patients consume adequate amounts of dietary fibre, this reduces intestinal transit time of food, thereby reducing carcinogen exposure [7,29]. Altogether, the evidence suggests that colorectal cancer patients who consume 22–24 g of fibre per day may reduce their risk of all-cause and colorectal cancer-specific mortality. In addition, it can be suggested that colorectal cancer patients consider replacing refined grains with whole grains as much as possible to help increase their fibre intake and decrease the risk of cancer recurrence.

### 3.3. Legumes and Soy

Legumes are an important component of PBDs and include beans, lentils, chickpeas, peas, and soybeans. They are a rich source of protein, fibre, vitamins, and minerals. There are few studies that have examined legumes solely in relation to cancer-related mortality and recurrence; however, the consumption of legumes provides prebiotic fibre, which has been demonstrated to induce changes in the gut microbiome [30]. Pre-clinical studies have shown the potential for the gut microbiome to influence anti-tumour immunity [31].

A single-centre 16-week randomized crossover study by Zhang et al. assessed the addition of one cup of navy beans in overweight or obese patients with a history of colorectal cancer or pre-cancerous polyps [32]. The addition of 1 cup of beans resulted in improved diversity and composition of the gut microbiome, including a reduction in pathogenic bacteria, as well as the modulation of immune and inflammatory biomarkers. The clinical significance of these changes to the microbiome and immune regulators requires further clarification, but some of the biomarkers, such as FGF-19, have been linked to metabolic diseases. This is relevant because there is ongoing evidence for an association between metabolic disorders like diabetes and cancer [32,33,34].

The question of soy consumption in relation to breast cancer prognosis is also important to address, given the controversy and concerns about the relationship between the estrogen-like properties of soy isoflavones and breast cancer. Current research shows that soy constituents may actually exhibit anti-estrogenic and other anti-cancer properties and are associated with a lower risk of developing breast cancer [35]. A systematic review and meta-analysis on the impact of soy and isoflavone consumption on breast cancer outcomes performed by Qiu et al. did not show a significant association between post-diagnosis soy intake and overall survival or breast cancer-specific mortality [36]. A prospective cohort study by Nechuta et al. took into account cultural differences in soy consumption by following 9514 breast cancer survivors from both the US and China. At a 7-year follow-up, post-diagnosis daily consumption of greater than or equal to 10 mg of isoflavones was associated with a 25% relative reduction in the risk of recurrence compared with women consuming less than 4 mg of isoflavones per day [35]. In women with ER-negative tumours, a stronger inverse association of soy isoflavone intake and recurrence was observed [35]. In a large multi-ethnic cohort study including 6235 women with breast cancer by Zhang et al., at a mean follow-up of 9 years, patients with the highest versus lowest intake of isoflavone had a 21% reduction in the risk of all-cause mortality. This reduction in the risk of all-cause mortality was limited to women with hormone receptor-negative tumours [37]. Hence, for patients with early-stage breast cancer, the consumption of soy foods within a PBD may decrease the risk of all-cause mortality and breast cancer recurrence.

### 3.4. Nuts and Seeds

Nuts and seeds are another component of a PBD, which are a source of healthy fats, protein, and fibre. A Shanghai-based cohort study followed 3449 breast cancer survivors for 10 years post-diagnosis and showed that nut consumption was positively associated with disease-free and overall survival in a dose-dependent manner. In this study, patients consuming greater than or equal to 17 g of nuts per week had improved disease-free and overall survival compared with non-consumers. These associations did not differ between different types of nuts [38].

In patients with non-metastatic prostate cancer, a large prospective cohort study demonstrated that post-diagnostic consumption of nuts, greater than or equal to five servings per week, was associated with a 34% lower relative risk of overall mortality compared to patients who consumed less than one serving per month [39].

Lastly, in a prospective observational study of 826 patients with stage III colon cancer, consuming two or more servings per week of tree nuts was associated with significantly improved disease-free and overall survival compared with non-consumers [40]. In regard to quantifiable guidance for patients, the Canada Food Guide recommends approximately one-quarter cup of nuts and seeds each per day as a single-serving size, or between 30 and 50 g, which would more than meet these minimum quantities [41]. However, it is unclear whether the daily serving recommendation of nuts and seeds by the Canada Food Guide would provide additional cancer control benefits based on the current evidence.

### 3.5. Red and Processed Meats

As previously mentioned, a plant-based diet does not inherently require the complete elimination of animal products but lends itself to incorporating many of the recommendations above. However, we thought it helpful to address recommendations regarding red and processed meats in cancer survivors, specifically those with CRC.

One narrative review by Zheng et al. in 2022 looked at different dietary patterns for CRC survivors [42]. They noted one epidemiological investigation relating red meat and processed meat consumption to global CRC deaths, as well as a prospective observational study that showed a more Western dietary pattern high in red and processed meat post-diagnosis being associated with worse disease-free survival and overall survival in patients with early-stage CRC [42,43,44]. They also mentioned that none of the studies compared the consumption of raw versus cooked meats, which vary between cultures [42]. In a prospective cohort study of colorectal cancer survivors, post-diagnosis plant-based diet index scores were significantly inversely associated with all-cause mortality [45]. Similarly, the ACS guidelines reported that multiple observational studies have demonstrated that a Western diet higher in processed meat is associated with an increased risk of colorectal cancer recurrence and prostate cancer-specific mortality [4,46].

In general, while there are no specific quantities that can be targeted, providers can suggest that CRC and prostate cancer patients consider minimizing red and processed meat intake as much as possible and consider replacing these foods with some of the categories described above.

## 4. Environmental Co-Benefits

There is also a large component of environmental co-benefits to the consumption of plant-based diets, which we thought pertinent to briefly discuss given ASCO’s recent 2024 policy statement on the impact of climate change on the delivery of oncology care [47].

The agricultural industry is responsible for approximately one-quarter of greenhouse gas (GHG) emissions, with meat and dairy livestock responsible for the majority [48,49,50,51]. Cattle feed crops are a major contributor to direct water consumption, while the health-harming pollution from ammonia produced by and for the maintenance of livestock was linked to approximately 12,000 premature deaths in the United States in 2018 alone [48]. The EAT–Lancet Commission on healthy, sustainable, and just food systems, published in 2025, sets out global targets to ensure sustainable food production for our planet [50]. The Commission specifically highlighted major concerns regarding GHG emissions, loss of biodiversity, fresh water consumption, and environmental disruption related to our current food systems [50]. By shifting our societal perspective of dietary health and its relationship to health outcomes, this may also help in shifting our environmental landscape. The Commission specifically recommended shifting to a plant-predominant diet, rich in whole grains, fruits, vegetables, nuts, and legumes, for a more sustainable ecological footprint. This eating pattern is described as the Planetary Health Diet (PHD) [50]. A recent prospective cohort study published in 2025 by Chen et al. showed that higher adherence to Eat Lancet’s Planetary Health Diet was associated with reduced all-cause and cancer-specific mortality in cancer survivors [52]. Chen’s study also showed that higher adherence to the PHD was correlated with lower systemic inflammation [52]. Inflammation may play a role in facilitating favourable conditions for the proliferation of malignant cells and angiogenesis [53]. Hence, closer adherence to a plant-based eating pattern, like the Planetary Health Diet, may improve mortality outcomes for cancer survivors through reductions in inflammation [52,53].

Models assessing the impact of vegan, vegetarian, pescatarian, and flexitarian diets demonstrated that plant-based foods cause fewer adverse environmental effects per serving than animal-sourced foods [50,54,55]. Springmann et al. defined a flexitarian diet as no processed meat, a maximum of one serving of red meat per week, and predominantly plant-based foods. A pescatarian diet replaced meat consumption with two-thirds of fish and seafood, and one-third of fruits and vegetables [54]. Within both Springmann’s model and the model used in Scarborough et al., vegan and vegetarian diets had the greatest reduction in GHG emissions [54,55]. Consequently, increasing PBD consumption could lead to a reduction in emissions of up to 80% by the year 2030 [54]. Springmann’s model also highlighted that reduced GHG emissions associated with greater PBD consumption are observed most in high-income countries [54].

Overall, a PBD could have benefits for cancer survivors and also contribute to the improved sustainability of our planet, which could thereby reduce the adverse health effects associated with climate change [50].

## 5. Brief Summary

Based on the evidence outlined above, we provide the following summary of dietary guidance for cancer survivors, highlighting key strategies for promoting overall health and reducing the risk of recurrence.

### 5.1. General Dietary Guidelines

When patients ask about what to eat after a cancer diagnosis, based on limited evidence, it can be suggested that they consider a diet that is rich in fibre and plant-based foods, including whole grains, nuts, seeds, vegetables, fruits, legumes, and soy foods. Additionally, they can consider minimizing their intake of red and processed meats, as well as refined grains, to possibly lower the risk of all-cause mortality [6,7]. This aligns with existing guidance from the CCS and ACS [3,4].

Key studies informing this review are included in Appendix A.

Cancer-specific dietary guidance is summarized in Table 1.

### 5.2. Environmental and Institutional Considerations

Beyond individual dietary choices, acknowledging the environmental co-benefits of plant-based diets is crucial. These discussions can take place at both the patient education level and within larger institutional frameworks. At the institutional level, advocating for plant-forward catering at department rounds, lectures, medical conferences can help promote change. In addition, offering more plant-based food options within hospital cafeterias and on inpatient food menus can also increase hospital staff and patient awareness. This plant-forward approach can be applied in various cultural and socioeconomic contexts [50,54]. These interventions can be paired with procurement from local food systems and strategies to limit food waste to maximize impact.

While shifting perspectives on dietary habits can be challenging, fostering conversations about the benefits of plant-based eating and raising awareness of the broader health impacts of climate change are important steps forward. Table 2 includes resources for patients, providers, and health systems.

### 5.3. Limitations

This is a literature review that used narrative reporting to summarize the best current evidence on plant-based dietary patterns and cancer outcomes in post-treatment patients. We recognize that the evidence presented is mostly small studies that have not been rigorously validated. We acknowledge the inherent bias in our selection of the studies discussed above, as we worked within the confines of existing cancer society guidelines that recommend plant-based diets. We did not systematically evaluate the quality of each study in our search within the parameters of this review.

The data itself also has significant limitations. The evidence behind dietary guidance is primarily from cohort studies, which, while valuable, provide weaker evidence than randomized controlled trials (RCTs). Although these observational studies are helpful for establishing an association between dietary patterns and cancer outcomes, they do not allow us to conclude whether there is a causal relationship between dietary patterns and cancer outcomes. In addition, most of the data in observational studies is collected through food frequency questionnaires, which rely on patient recall and are inherently subjective. This raises concerns about the accuracy of dietary assessments. Another limitation is that many observational studies assess dietary patterns only prior to diagnosis, without accounting for any dietary modifications patients may have made post-diagnosis as part of their survivorship journey. Furthermore, as outlined above, quantities of different dietary components are variable between studies. Validation of this data would require large randomized controlled trials, which is challenging. Unlike drug studies, patient diets are difficult to control for and survival outcomes can be influenced by multiple other factors.

Due to the low quality of evidence presented, we have specifically not made recommendations, but instead offered dietary guidance that providers can suggest to patients. This is in keeping with guideline practices used by the World Cancer Research Fund Cancer Update Program [56].

There are also significant cultural differences in the types of foods consumed, which are only considered in a few studies. Although general guidance may be applicable across cultural contexts, we acknowledge that cancer survivors may have challenges with specific guidance, including cultural appropriateness, household member diets, and food insecurity.

Lastly, we focused on breast, colorectal, and prostate cancer survivors in this review; however, we hope that in the future, as general attitudes tend to shift towards patient-centred and holistic approaches to care, further studies will continue to explore dietary patterns and their associations with outcomes in different types of cancers.

## Figures and Tables

**Table 1:** Cancer specific dietary guidance that may improve cancer outcomes.

Type of Cancer	Dietary Guidance
Breast Cancer	Consuming a small daily serving of soy is safe and may reduce the risk of recurrence. Higher fibre intake may decrease the risk of breast cancer-specific and all-cause mortality. Adding a small serving of ¼ cup of nuts once per week may improve disease-free survival.
Colorectal cancer	Consuming higher amounts of processed meat may increase the risk of colorectal cancer recurrence Increasing fibre to 22–24 g/day may reduce the risk of disease-specific mortality. ○ High-fibre fruits like berries can also be beneficial if they are accessible. ○ Chickpeas, beans, oats, and cruciferous vegetables (e.g., broccoli and brussels sprouts) are affordable options. Substituting one serving of refined grains for one serving of whole grains each day is associated with improvements in disease-free survival. Consuming 2 or more servings per week of tree nuts may improve disease-free and overall survival.
Prostate cancer	Consuming a more Western diet and/or high amounts of processed meat may increase the risk of prostate cancer mortality. For non-metastatic prostate cancer, a higher consumption of nuts may reduce overall mortality risk. For those with high-risk disease, incorporating a daily serving of tomatoes, which are rich in lycopene, may reduce the risk of prostate cancer mortality.

**Table 2:** Quick resources.

Topic	Resources
Switching to a plant-based diet	Healthlink BC—General Plant-based eating patterns: https://www.healthlinkbc.ca/living-well/food-and-nutrition/food-and-your-health/plant-based-eating (accessed on 27 December 2025) Healthlink BC—Plant-based eating guidelines: https://www.healthlinkbc.ca/healthlinkbc-files/plant-based-eating-guidelines (accessed on 27 December 2025) Nourish—supporting plant-forward health care food systems: https://nourishleadership.ca/ (accessed on 27 December 2025) American Institute for Cancer Research—How to Get Enough Protein on a Plant-Based Diet: https://www.aicr.org/resources/blog/how-to-get-enough-protein-on-a-plant-based-diet/ (accessed on 27 December 2025)
Impact of climate change on oncology care	American Society of Clinical Oncology (ASCO)—Climate Change and Cancer Care: A Policy Statement from ASCO (2023): https://ascopubs.org/doi/10.1200/OP.23.00637 (accessed on 27 December 2025)
Improving sustainability in healthcare	European Society For Medical Oncology (ESMO)—ESMO Climate Change Task Force: https://www.esmo.org/about-esmo/organisational-structure/esmo-task-forces/climate-change-task-force (accessed on 27 December 2025) Climate Change: Why Oncologists Need to get Involved: https://www.nature.com/articles/s44276-023-00023-9#Sec4 (accessed on 27 December 2025)

## Data Availability

No new data were created or analyzed in this study. Data sharing is not applicable to this article.

## Appendix A

**Table A1:** Key studies informing narrative review.

Title	Author	Publication Year	Study Design Follow-Up	Cancer Type	Sample Size	Dietary Exposure	Dietary Scoring System	Survival Endpoints
Planetary health diet index and mortality among US cancer survivors: mediating roles of systemic immune-inflammation index and neutrophil-to-lymphocyte ratio.	Chen H, et al. [52]	2025	Prospective Cohort Study; FU 10.4 years (median)	All cancers	3442 patients	Planetary Health Diet (PHD) introduced by the EAT-Lancet Commission in 2019	Planetary Health Diet Index (PHDI)	All-cause mortality, cancer-specific mortality, non-cancer mortality
Association between Dietary Fiber Intake and Mortality among Colorectal Cancer Survivors: Results from the Newfoundland Familial Colorectal Cancer Cohort Study and a Meta-Analysis of Prospective Studies.	Zhao J, et al. [27]	2022	Prospect Cohort Study; FU 7–11 years	Colorectal Cancer	504 patients	Dietary fibre	Food Frequency Questionnaire (FFQ)	All-cause mortality
Nut Consumption in Association with Overall Mortality and Recurrence/Disease Specific Mortality Among Long-Term Breast Cancer Survivors.	Wang C, et al. [38]	2022	Prospective Cohort Study; FU 8.27 years (median)	Breast Cancer (at least 5-years post-diagnosis)	3449 patients	Nut consumption, including peanuts, walnuts, and other nuts. Soy foods, meat, fish, and cruciferous vegetables.	Structured Questionnaire on intake, then food frequeny questionnaire (FFQ) at follow-up	All-cause mortality, disease-free survival
Postdiagnostic Fruit and Vegetable Consumption and Breast Cancer Survival: Prospective Analyses in the Nurses’ Health Studies.	Farvid MS, et al. [20]	2021	Prospective Cohort Study; FU 30 years	Breast Cancer (Stage I–III)	8927 patients	Fruits, vegetables, dietary fibre, total fat, animal fat, alcohol	Food Frequency Questionnaire (FFQ)	All-cause mortality, breast-cancer specific mortality
Post-diagnostic reliance on plant-compared with animal-based foods and all-cause mortality in omnivorous long-term colorectal cancer survivors.	Ratjen I, et al. [45]	2021	Prospective Cohort Study; FU 7 years (median)	Colorectal Cancer	1404 patients	Healthful plant-based vs. unhealthful plant-based diet	Food Frequency Questionnaire (FFQ) to calculate plant-based diet indices	All-cause mortality
Association of Survival With Adherence to the American Cancer Society Nutrition and Physical Activity Guidelines for Cancer Survivors After Colon Cancer Diagnosis.	Van Blarigan EL, et al. [15]	2018	Prospective Cohort Study; FU 7 year (median)	Colorectal Cancer (Stage III)	992 patients	Fruits, vegetables, whole grains, refined grains, red and processed meats, alcohol	Food Frequency Questionnaire (FFQ) incorporated into American Cancer Society (ACS) Guidelines Score	All-cause mortality, disease-free and recurrence-free survival
Fiber Intake and Survival After Colorectal Cancer Diagnosis.	Song M, et al. [26]	2018	Prospective Cohort Study; FU 8 years (median)	Colorectal Cancer (Stage I–III)	1575 patients	Total fibre, different sources of fibre (cereals, vegetables, fruits), whole grains	Food Frequency Questionnaire (FFQ)	All-cause mortality, CRC-specific mortality
Grain Intake and Clinical Outcome in Stage III Colon Cancer: Results	Brown JC, et al. [28]	2018	Prospective Cohort Study; FU 7.3 years (median)	Colorectal Cancer (Stage III)	1024 patients	Refined and whole grains	Food Frequency Questionnaire (FFQ)	Disease Free Survival
Nut Consumption and Survival in Patients With Stage III Colon Cancer: Results From CALGB 89803 (Alliance).	Fadelu T, et al. [40]	2018	Prospective Cohort Study; FU 6.5 years (median)	Colorectal Cancer (Stage III)	826 patients	Nut consumption	Semi-quantitative food frequency questionnaire (FFQ)	Disease free survivial, recurrence-free survival, all-cause mortality
Dietary isoflavone intake and all-cause mortality in breast cancer survivors: The Breast Cancer Family Registry. Cancer.	Zhang FF, et al. [37]	2017	Prospective Cohort Study; FU 9.4 years (median)	Breast Cancer	6235 patients	Soy isoflavone intake	Food Frequency Questionnaire (FFQ)	All-cause mortality
Lycopene, tomato products and prostate cancer-specific mortality among men diagnosed with nonmetastatic prostate cancer in the Cancer Prevention Study II Nutrition Cohort. Int J Cancer.	Wang Y, et al. [22]	2016	Prospective Cohort Study; FU 15 years	Prostate Cancer	8898 patients	Lycopene and tomato products	Modified brief Block Food Frequency Questionnaire (FFQ) and modified Willett FFQ	Prostate-cancer specific mortality
Nut consumption and prostate cancer risk and mortality.	Wang W, et al. [39]	2016	Prospective Cohort Study; FU 26 years	Prostate Cancer	4346 patients	Nut consumption	Semi-quantitative food frequency questionnaire (FFQ)	Prostate-cancer specific mortality
Soy food intake after diagnosis of breast cancer and survival: an in-depth analysis of combined evidence from cohort studies of US and Chinese women.	Nechuta SJ, et al. [35]	2012	Prospective Cohort Study; FU 7.4 years (mean)	Breast Cancer	9514 patients	Soy isoflavone intake	Food Frequency Questionnaire (FFQ)	All-cause mortality, breast-cancer specific mortality
